# Evaluating the Quality of Asynchronous Versus Synchronous Virtual Care in Patients With Erectile Dysfunction: Retrospective Cohort Study

**DOI:** 10.2196/32126

**Published:** 2022-01-13

**Authors:** Lauren Broffman, Melynda Barnes, Kevin Stern, Amy Westergren

**Affiliations:** 1 Ro New York, NY United States

**Keywords:** telehealth, medical informatics, side effect, virtual health, platform, medication, sync, outcome, adverse event, drug, electronic health record, treatment, erectile dysfunction

## Abstract

**Background:**

Asynchronous health care encounters are becoming an increasingly mainstream form of telehealth. While synchronous phone or video visits have become more widely accepted, US policymakers and other key health care stakeholders have been hesitant to fully embrace asynchronous diagnosis and treatment. This is particularly true in the context of direct-to-consumer (DTC) platforms, where encounters are patient-initiated and there is no preestablished relationship with a provider. This hesitation is compounded by limited research comparing outcomes between asynchronous and synchronous care, especially in the DTC context.

**Objective:**

The purpose of this study was to explore whether asynchronous care leads to different patient outcomes in the form of medication-related adverse events when compared to synchronous virtual care.

**Methods:**

Using 10,000 randomly sampled patient records from a prominent US-based DTC platform, we analyzed the rates of patient-reported side effects from commonly prescribed erectile dysfunction medications and compared these rates across modalities of treatment.

**Results:**

Asynchronous care resulted in lower but nonsignificant differences in the rates of the reported drug-related side effects compared to synchronous treatment.

**Conclusions:**

In some circumstances, such as treatment for erectile dysfunction, asynchronous care can offer the same level of safety in prescribing when compared to synchronous care. More research is needed to evaluate the safety of asynchronous care across a wider set of conditions and measures.

## Introduction

Asynchronous technology is becoming an increasingly common component of health care delivery following the explosion of telehealth in the wake of the COVID-19 pandemic [1]. This technology serves as both a complement to synchronous (ie, real time) encounters and, increasingly, as a replacement for other modes of diagnostic and treatment-related interactions between patients and providers.

Asynchronous care relies on “store-and-forward” technology where patients provide necessary health information that is transmitted to providers, who then make a diagnosis and design a treatment plan on secure web-based platforms (sometimes called electronic or e-visits). The growing popularity of asynchronous care reflects the advantages it confers by removing the need for provider and patient co-availability, in which case treatment can be accessed and delivered at everyone’s convenience [2]. Research shows patients will take advantage of timing flexibility to engage in care during evenings and weekends [3] and that both patient and provider satisfaction with asynchronous care are high [4,5]. Evidence also suggests that asynchronous care could help mitigate access barriers associated with the “digital divide,” allowing patients to access services even in areas where high-speed internet required to support video calls is not widely available [6,7].

Though the pandemic led to regulatory revisions permitting reimbursement for asynchronous care [8], policymakers and practitioners have maintained reservations, particularly in direct-to-consumer (DTC) scenarios where care is commonly delivered asynchronously but patients and providers do not have a preexisting relationship [2,9]. This hesitation is justified given the limited evidence on whether telemedicine can lead to inappropriate prescribing and increased risk of failing to identify factors that might contribute to increased side effects [10]. There is also scant evidence on the health and quality outcome implications for e-visits compared to in-person or other forms of virtual care [11]. For example, one recent randomized controlled trial comparing asynchronous and synchronous telepsychiatry showed similar patient outcomes across modalities [12]. However, another study comparing these modalities found that asynchronous care might create conditions that could negatively impact patient safety: providers adopted different prescribing behaviors depending on the modality of treatment for otherwise similar patients [13]. A recent literature review identified only 19 studies that quantitatively evaluated e-visits and concluded that while they appeared to result in similar health outcomes as compared to in-person care, evidence on quality outcomes is mixed, and there were no included studies comparing them to telephone or video visits [8].

The popularity of DTC companies that rely on asynchronous care is increasing, and the sentiment that they are “here to stay” has led researchers to explicitly highlight the need for published data rates on adverse events on these platforms [10,14]. The purpose of this study was thus to explore whether asynchronous care leads to different patient outcomes in the form of medication-related adverse events when compared to synchronous virtual care using a sample of 10,000 men undergoing treatment for erectile dysfunction (ED), a commonly treated condition on DTC platforms.

## Methods

### Study Design

We build on our previously published research regarding the rates of side effects experienced by DTC patients being treated for ED on a platform that offers synchronous and asynchronous telehealth services for a variety of health conditions [15]. This study was approved by the Biomedical Research Alliance of New York Institutional Review Board.

### Study Sample

In the original study, we analyzed all electronic health records for 10,000 randomly selected ED patients being treated on a single DTC platform starting sometime in 2018. Each patient was prescribed either sildenafil or tadalafil, two generic PDE-5 (phosphodiesterase type 5) inhibitor medications, which serve as first-line treatments for erectile dysfunction [16].

### Study Variables

To compare the differences across treatment modalities, we take advantage of the variations in state laws that dictate whether a patient can be treated asynchronously or that they must engage with the provider via phone or video call. Patients who elect to be treated asynchronously might be systematically different from patients who opt to be treated synchronously, introducing bias (eg, patients who prefer asynchronous treatment might be younger and less likely to have age-related conditions that make them more susceptible to adverse events). However, laws regulating the application of asynchronous treatment vary by state; therefore, a portion of the patients in the sample were required to engage in synchronous information exchange with their provider in order to receive treatment. For the purpose of this study, we assumed population homogeneity and did not consider residence in a different state as a factor that might affect either the rate of side effects or the rate at which those side effects are reported. This mitigates any confounding that might have been introduced by patients self-selecting a given visit modality.

State residency was determined using the patient’s mailing address that was provided for medication shipments. We then defined asynchronously treated patients as those who reside in a state where asynchronous treatment is permitted per state law. Patients residing in these states completed an asynchronous online visit that was reviewed by a provider who then engaged with a patient via chat to discuss the diagnosis and treatment plan and dispensed a prescription. By contrast, we defined synchronously treated patients as those who reside in states where either a phone or video consultation is required by state law in order for a prescription to be dispensed. Patients in these states also completed an online visit, but then scheduled and completed a phone or video call with a provider before a prescription was dispensed. We note that patients who reside in states where asynchronous treatment is permitted can elect to have a phone or video consult before treatment commences; however, instances of pretreatment modality switching are highly uncommon on the platform and did not occur in this sample. A small number of asynchronously treated patients had phone or video conversations with providers after the treatment began, but we did not disqualify them from the asynchronous group because the synchronous interaction did not occur before a prescription was dispensed.

We defined an adverse event as an instance when a patient reported a medication side effect to their provider after beginning treatment on the DTC platform. To determine the rates of reported side effects in the original study, a research team of medical doctors and scientists reviewed a comprehensive set of patient records that included interactions starting from initial treatment sometime in 2018 through September 2019 and flagged any instance in which a patient contacted their provider to report a side effect [15]. To accomplish this, the team compiled a comprehensive list of side effects based on published literature [17-19] and created a set of colloquial search terms. For example, in addition to “flushing,” we included words like “hot” and “red.” The records were searched against the terms by calculating the Levenshtein distance using the ‘stringdist’ package by R (R Foundation for Statistical Computing) to allow us to capture key terms that were spelled incorrectly or phrased slightly differently than the list verbiage [20]. The records were sorted in order of calculated distance and manually reviewed for categorization until matches were exhausted. The identified side effects were then broken down by medication and type [15]. For quality control purposes, a subset of patient records reported to contain no side effects was manually reviewed to ensure that the search procedure did not systematically overlook any adverse events.

Because there is some evidence indicating that the risk of medication-related adverse events increases with age [21], patient age was extracted from their electronic health records and included as a control variable.

### Analysis

Each record was also assigned a categorical indicator for whether a patient had a synchronous (either a phone or video call) interaction with a provider before being prescribed medication or that diagnosis and treatment selection occurred asynchronously. We then compared the rates of any side effect reported by patients treated either asynchronously or synchronously. The data were modeled using generalized linear models, and analysis was carried out using R, version 4.03.

## Results

The average age of patients whose records were included in the sample was 44.8 years (SD 12.1). The majority (78% [n=7850]) were treated asynchronously. Overall, patient-reported side effects were rare; less than 2% (n=137) of patients reported experiencing any side effect. In concordance with definitions used by McMurray et al [22] and Montorsi et al [19], we determined that no serious adverse events, such as myocardial infarction, vision or hearing loss, or cerebrovascular accident, were reported. Across modalities, 1.12% (n=113) of synchronously treated patients reported experiencing a side effect compared to 1.44% (n=24) of asynchronously treated patients (Table 1). There were no notable differences across type and distribution of mild side effects.

**Table 1:** Rates of reported side effects by modality.

Side effects	Synchronous (n=2150)	Asynchronous (n=7850)
Any side effect, n (%)	24 (1.12)	113 (1.44)
Headache, n (%)	10 (0.47)	56 (0.71)
Dizziness, n (%)	0 (0)	3 (0.04)
Flushing, n (%)	2 (0.09)	31 (0.39)
Congestion, n (%)	12 (0.56)	17 (0.22)
Dyspepsia, n (%)	7 (0.33)	10 (0.13)
Back pain, n (%)	0 (0)	6 (0.08)
Blurry vision, n (%)	2 (0.09)	8 (0.10)
Other, n (%)	2 (0.09)	7 (0.09)

We employed 2 different generalized linear models to determine whether the difference in side effect rates for asynchronously or synchronously treated patients, however small, was statistically significant. The first model was a standard logit model with the dichotomous indicator for whether a patient reported experiencing a side effect as the outcome. We found that asynchronous patients were around 20% less likely to report experiencing a side effect (odds ratio 0.77; *P*=.26), but this difference was not significant.

Because fewer than 2% (n=137) of the patients reported experiencing a side effect, standard logistic regression might bias results toward zero by underestimating the probability of a side effect occurring even with the large sample size [23]. As a sensitivity check, we used a penalized likelihood estimator (the Firth method) [24]. The results in the penalized regression were similar, with asynchronous patients also around 15% less likely to report experiencing a side effect (odds ratio 0.84; *P*=.28), but this difference was not significant.

**Table 2:** Generalized linear model results.

	Standard logit					Binomial response			
	OR^a^	SE	Z	Pr(>|z|)	OR		SE	Z	Pr(>|z|)
Intercept	0.02***	0.09	-44.60	.000	0.01		0.11	-38.75	<.001
Asynchronous	0.77	0.23	-1.14	.26	0.84		0.16	-1.077	.28

^a^OR: odds ratio

***P<.001

## Discussion

The recent widespread adoption of telehealth as an acceptable treatment modality and the potential expansion of asynchronous care have prompted deeper exploration of the downstream effects. Research that focuses on telehealth treatment outcomes and the reported side effects can facilitate defining standards, improving quality of care, and identifying opportunities for expanding treatment access to more patients and for more conditions.

Our exploration of the rates of reported side effects among people receiving synchronous versus asynchronous care offers a chance to explore any unexpected downstream effects these modalities might have on patient safety and treatment outcomes. Significantly higher rates of side effects for asynchronous treatment might suggest that removing a real time interaction between patient and provider precludes the necessary information gathering in order to make a clinically appropriate assessment of whether a patient should receive medication. Alternatively, significantly lower rates of side effects from asynchronous treatment might indicate that removing the real time patient-provider interaction precludes the necessary rapport building that would encourage patients to contact their provider in case of an adverse event. Overall, we found that though the odds of reporting a side effect were lower for asynchronous patients, they did not significantly differ from the odds among synchronous patients. These results suggest that in this context (DTC treatment for ED), asynchronous care via patient-initiated encounter does not unduly prevent patients from reporting an adverse event, nor does it result in any other notable differences in adverse events when compared to patients who received treatment after a phone or video call. Our findings corroborate those of the handful of other studies in different areas of medicine in which comparisons of patient outcomes across telehealth modalities yielded no disproportionate rates of adverse events [12,25].

Copious research is required to continue to evaluate the safety of asynchronous care across different conditions and circumstances; however, current evidence suggests that lawmakers and practitioners should continue to consider facilitating its adoption with a prudent approach to implementation that takes into account specific circumstances where asynchronous care is safe and appropriate. These considerations could also factor in circumstances under which certain sites of patients are experiencing access barriers (such as broadband availability) that prevent them from using video technology due to bandwidth limitations [26], or stigma-related barriers that prevent patients from seeking care that requires a face-to-face interaction.

Acknowledging asynchronous care’s departure from the traditional practice of medicine while simultaneously embracing its benefits could entail the design and execution of programs that offer training for providers on best practices for care that relies on store-and-forward technology [27,28]. Asynchronous-specific quality metrics could be refined and tracked to ensure that care is of consistently high quality [7]. Both training and metrics could include mitigating and tracking unsafe prescribing practices, respectively.

There are several limitations to this study. First, the results are specific to a single DTC platform and might not be generalizable across all DTC platforms and patients. For example, structured online intake forms and requested patient data vary by DTC platform, thus introducing variation in the evidence-based nature, breadth, and quality of information presented to providers for clinical evaluation. Second, the record lookback window in the sample ended in 2019; COVID-19 has likely introduced changes in the DTC patient population. For example, patients who had not previously considered seeking treatment via telehealth might have been prompted by the sudden and unexpected inability to get in-person care. Lastly, the results also might not be generalizable beyond the condition and medications studied. More research needs to be conducted to determine whether the rates of side effects differ across modalities for other conditions that are commonly treated on DTC platforms, especially as these platforms continue to expand their treatment offerings to provide a more comprehensive suite of services.

Limitations notwithstanding, this study represents an important step toward a more nuanced approach to evaluating the quality of care delivered via telehealth. As telehealth demonstrated its value during the global pandemic and is becoming an increasingly normalized form of care, research needs to evolve beyond comparisons to in-person care toward identifying the most ideal formats, processes, and approaches for collecting relevant clinical information and safely treating and communicating with patients within the suite of virtual options.

## Abbreviations

DTC: direct-to-consumer
ED: erectile dysfunction
PDE-5: phosphodiesterase type 5
