# Correction: Evaluating the Quality of Asynchronous Versus Synchronous Virtual Care in Patients With Erectile Dysfunction: Retrospective Cohort Study

**DOI:** 10.2196/41121

**Published:** 2022-07-27

**Authors:** Lauren Broffman, Melynda Barnes, Kevin Stern, Amy Westergren

**Affiliations:** 1 Ro New York, NY United States

In “Evaluating the Quality of Asynchronous Versus Synchronous Virtual Care in Patients With Erectile Dysfunction: Retrospective Cohort Study” (JMIR Form Res 2022;6(1):e32126), the authors noted the following corrections:

1. In Table 1, the values for each side effect - headache, dizziness, flushing, congestion, dyspepsia, back pain, and blurry vision - were mislabeled. Values categorized as ‘asynchronous’ should have been labeled ‘synchronous’ and vice versa. No values were incorrectly referenced in the text of the article and this error does not impact the overall interpretation of results.

2. In the original paper, the following sentence was present in the *Discussion* section:

The recent widespread adoption of telehealth as an acceptable treatment modality and the exploration for potential expansion of traditional methods outside the scope of synchronous care have prompted deeper exploration of the downstream effects of these approaches to care distribution.

This has been changed to:

The recent widespread adoption of telehealth as an acceptable treatment modality and the potential expansion of asynchronous care have prompted deeper exploration of the downstream effects.

3. The phone number of the corresponding author, Lauren Broffman, has been updated to 1 888 798 8686.

**Table 1:** Rates of reported side effects by modality.

Side effects	Synchronous (n=2150)	Asynchronous (n=7850)
Any side effect, n (%)	24 (1.12)	113 (1.44)
Headache, n (%)	10 (0.47)	56 (0.71)
Dizziness, n (%)	0 (0)	3 (0.04)
Flushing, n (%)	2 (0.09)	31 (0.39)
Congestion, n (%)	12 (0.56)	17 (0.22)
Dyspepsia, n (%)	7 (0.33)	10 (0.13)
Back pain, n (%)	0 (0)	6 (0.08)
Blurry vision, n (%)	2 (0.09)	8 (0.10)
Other, n (%)	2 (0.09)	7 (0.09)

The correction will appear in the online version of the paper on the JMIR Publications website on July 27, 2022, together with the publication of this correction notice. Because this was made after submission to PubMed, PubMed Central, and other full-text repositories, the corrected article has also been resubmitted to those repositories.

